# BushenHuoxue decoction suppresses M1 macrophage polarization and prevents LPS induced inflammatory bone loss by activating AMPK pathway

**DOI:** 10.1016/j.heliyon.2023.e15583

**Published:** 2023-04-19

**Authors:** Shuangshuang Chen, Lihong Tao, Feng Zhu, Zhifang Wang, Qi Zhuang, Yajun Li, Yunshang Yang, Chengcheng Feng, Haiwei Shi, Jiandong Shi, Like Zhu, Long Xiao, Dechun Geng, Zhirong Wang

**Affiliations:** a Translational Medical Innovation Center, Zhangjiagang TCM Hospital Affiliated to Nanjing University of Chinese Medicine, Zhangjiagang, 215600, China; b Department of Rheumatology, Zhangjiagang TCM Hospital Affiliated to Nanjing University of Chinese Medicine, Zhangjiagang, 215600, China; c Department of Orthopedics, The First Affiliated Hospital of Soochow University, Suzhou, 215006, China; d Department of Orthopedics, Zhangjiagang TCM Hospital Affiliated to Nanjing University of Chinese Medicine, Zhangjiagang, 215600, China

**Keywords:** Inflammatory bone loss, Bushen huoxue decoction, Inflammation, Macrophage polarization, Bone immune regulation

## Abstract

Abnormal bone metabolism and subsequence osteoporotic fractures are common complications of chronic inflammatory diseases. No effective treatment for these bone-related complications is available at present. The chronic inflammatory state in these diseases has been considered as a key factor of bone loss. Therefore, the combination of inflammation inhibition and bone loss suppression may be an important strategy for reducing bone damage associated with inflammatory diseases. Bushen Huoxue Decoction (BSHXD) is a traditional Chinese herbal compound that has demonstrated the ability to improve bone quality and increase bone density. However, the efficacy of BSHXD on inflammatory bone loss and its underlying mechanisms remain unclear. This study aimed to investigate whether BSHXD inhibits inflammatory bone loss in mice and its potential molecular mechanisms. In the present study, the effect of BSHXD on lipopolysaccharide (LPS)-induced M1 polarization of RAW264.7 macrophage and on local inflammatory bone loss model of mouse skull was determined. The results showed that after treating RAW264.7 cells with LPS for 24 h, the expression levels of IL-1β (39.42 ± 3.076 ng/L, p < 0.05), IL-6 (49.24 ± 1.766 mg/L, p < 0.05) and TNF-α (286.3 ± 27.12 ng/L, p < 0.05) were significantly increased. The addition of BSHXD decreased the expression levels of IL-1β, IL-6, and TNF-α to 31.55 ± 1.296 ng/L, 37.94 ± 0.8869 mg/L, and 196.4 ± 25.25 ng/L, respectively (p < 0.05). The results of immunofluorescence staining, Western blotting (WB) and flow cytometry indicated that the proportion of M1 macrophages in RAW264.7 cells treated with BSHXD for 24 h was significantly lower than that in the LPS group (13.36% ± 0.9829% VS 24.80% ± 4.619%, p < 0.05). The evidence from *in-vitro* experiments showed that the immunomodulatory ability of BSHXD may be associated with the activation of AMP-dependent protein kinase (AMPK) pathway in LPS-treated macrophages. In addition, the results of micro-CT, H&E staining, immunohistochemical staining and immunofluorescence staining of mouse skull further demonstrated that BSHXD treatment significantly alleviated LPS-induced local bone loss and inflammatory damage in mouse skull model. All results indicated that BSHXD significantly inhibited inflammatory factors release and M1 polarization of macrophage through AMPK signaling pathway. Therefore, BSHXD may be a promising drug for the treatment of inflammatory bone loss.

## 1 Introduction

Inflammatory bone loss refers to the increased bone resorption and decreased bone formation caused by the interaction of inflammatory cytokines [1] with osteocytes [2], during which inflammation becomes the main trigger of bone loss [3,4]. Inflammatory bone loss often occurs in chronic inflammatory diseases, such as rheumatoid arthritis (RA) [5,6], spondyloarthritis [7], chronic obstructive pulmonary disease, periodontitis [8], etc. Therefore, these diseases are prone to osteoporosis and fractures. Previous studies have shown that chronic inflammation [2] can lead to bone damage, erosion, resorption and further inhibit bone formation [9]. The underlying mechanism may be related to the complex interactions [10,11] between monocyte, macrophage [12] and osteoclast lineage [13].

In the inflammatory state, macrophages are polarized [14] to M1 macrophages and further aggravate the local inflammatory status. A large number of pro-inflammatory cytokines and receptor activator of nuclear factor kappa B ligand (RANKL) are released by M1 macrophage, which further activates the nuclear factor kappa-B (NF-κB) pathway, promotes osteoclast differentiation, increases bone resorption [15], and eventually leads to bone loss [[16], [17], [18]]. Therefore, the inflammatory process is closely related to M1 polarization of macrophages, and controlling this chronic inflammatory state is the key to alleviating inflammatory bone loss [11,19].

Previous studies have shown that modulating macrophage polarization inhibits the inflammatory microenvironment, thereby reducing bone loss. For example, the inhibition of LPS-stimulated M1 macrophage polarization attenuates periapical inflammation and cementum loss [20]. In another study, Isobavachalcone can inhibit M1 polarization of macrophages, thus treating osteoporosis [21]. Furthermore, Sirtuin 1 can suppress synovial inflammation in RA by reducing the number of local macrophages [22]. Therefore, inhibiting the M1 polarization of macrophages can reduce the local inflammatory level and may be an important direction and potential treatment for inflammatory bone loss.

According to traditional Chinese medicine (TCM), bone loss leaded to osteoporosis is considered as the manifestation of external manifestations of kidney deficiency. The function of kidney is closely related to the development and function of bone. The essence stored in the kidney can provide nutrition for bones, and help bone growth and repair, and enhance bone mass density [23]. A wealth of information indicates that the traditional Chinese medicine with the function of tonifying the kidney, promoting blood circulation and removing blood stasis usually has a significant effect in the treatment of osteoporosis [24]. BSHXD is a compound prescription of traditional Chinese medicine, which can alleviate joint pain [25], increase bone density and bone quality [26,27]. However, the mechanism of BSHXD action has not been elucidated.

The purpose of this study was to investigate the effect of BSHXD on LPS-induced M1 polarization of macrophages and related signaling pathways, so as to further understand the mechanism of BSHXD in the treatment of inflammatory bone loss. In this study, WB, IF and flow cytometry were used to evaluate the effect of BSHXD on M1 polarization and proinflammatory factor expression of macrophages in vitro. And further explore the relevant molecular mechanisms. In vivo experiment, LPS induced local inflammatory bone loss model of mouse skull was used to analyze the bone histomorphometry after BSHXD administration to evaluate the role of BSHXD in preventing bone loss.

## 2 Methods

### 2.1 Preparation of BushenHuoxue decoction

The main ingredients of BushenHuoxue Decoction used in this study are Astragalus membranaceus, Rehmannia glutinosa, Epimedium, Psoraleae, Bidentata, Angelica sinensis, Salvia miltiorrhiza, Peony and Pueraria, and the ratio is 4:3:2.4:2.4:3:3:3. All BSHXD Chinese medicinal materials from Beijing Tongrentang were used in this study and identified in the School of Pharmacy of Nanjing University of Traditional Chinese Medicine. The certified specimens of the above nine herbs were preserved in the Herbarium Center of Nanjing University of Chinese Medicine. The Chinese herbal medicine components of BSHXD were mixed in 1000 mL distilled water and soaked for 30min. The mixed medicine was boiled for more than 1 h and 300 mL of medicine juice was extracted for 2 times. The mixture was filtered and the filtrate was concentrated to the drug content of 2.6445 g/mL. In order to prepare BSHXD extract for in vitro cell studies, 48.8 mL (2.6445 g/mL) was freeze-dried with a lab-1A-50 E freeze dryer to obtain 2.15 g of freeze-dried powder for in vitro studies. BSHXD preparation was stored at −20 °C.

### 2.2 Qualitative analysis of the chemical profile in BSHXD using HPLC-HR-MS

The BSHXD with crude drug content at 1 g/mL was used in this study. Briefly, 0.6 mL of acetonitrile was added into a 1.5 mL tube which contains 0 .2 mL BSHXD, then the tube was vortexed for 10min and subject to centrifugation at 21,000 g for 10 min. The supernatant was collected for the LC-Q-TOF/MS analysis.

The LC-Q-TOF/MS was consisted of a Shimadzu UFLC 20ADXR system and a TripleTOF 5600 mass spectrometer. The LC conditions was as follows: a XBridge BEH C18 (2.1mm × 50 mm, 1.7 μm) column was used for separation and the column was maintained at 40 °C. 0.01% formic acid-water (A) and acetonitrile (B) were used as the mobile phase. The flow rate was 0.3 mL/min and a gradient elution program was used. The separated anlystes were then ionization by electrospray ionization (ESI) in positive or negative mode. And the parent ions and the product ions were collected by the TOF mass spectrometer. The MS1 *m*/*z* scan range was 50–1200, and *m*/*z* range used in information dependent acquisition mode for MS/MS was 50–1000.

### 2.3 Cell culture

RAW264.7 cells (FH0328) and THP-1 cells (FH0112) were purchased from FuHeng BioLog in Shanghai, which were used for in vitro experiment. In the RAW264.7 cells, DMEM medium (SH30022.01, Cytiva, Pittsburgh, USA) containing 100U/mL penicillin-streptomycin-amphotericin-amphotericin B (C100C8, NCM Biotech, SuZhou, China) and 10% fetal bovine serum (FBS, 16,140,071, Gibco, Rockville, USA) were used as culture medium. Cells were incubated in a 37 °C, 5% carbon dioxide incubator. In the THP-1 cells, RPMI1640 medium culture (A4192301, Gibco, Grand Island, USA) containing 10% FBS serum were used as culture medium. THP-1 monocytes are differentiated into macrophages by 24 h incubation in 1640 medium with 100 μM phorbol 12-myristate 13-acetate (PMA, P8139, Sigma-Aldrich, Missouri, USA). Then the medium was changed and 20 ng/mL IFN-γ (SRP3058, Sigma-Aldrich) and 10 pg/mL LPS (#8630, Sigma) were added into 1640 medium to induce M1-type macrophages.

### 2.4 Cell viability tests

CCK8 kit (CK04, Dojindo, ShangHai, China) was used to detect the toxic effect of BSHXD on RAW264.7 cells. 1 × 10^4^ cells were cultured in a 96-well plate. After the cells were attached, concentration gradient of BSHXD (0, 0.1 μg/mL, 1 μg/mL, 10 μg/mL, 50 μg/mL, 100 μg/mL, 500 μg/mL) were used to treatment cells. After 24 h incubation in the incubator, 10 μl of CCK8 solution were added to each well. After waiting for a certain time, we detected OD value with a microplate reader (BioTek, Vermont, USA) and calculated cell viability.

### 2.5 Enzyme-linked immunosorbent assay (ELISA)

The levels of inflammatory factors in cell supernatants were evaluated using standard ELISA kits (BOSTER, Wuhan, China). RAW264.7 cells (1 × 10^5^ cells/well) were treated with BSHXD 1 h before stimulated with 1 μg/mL LPS for 24 h. Cell culture medium was collected and centrifuged at 3000 rpm for 10min. Using a standard ELISA kit detected the supernatant. Optical density was determined by a microELISA plate reader at 450 nm.

### 2.6 Immunofluorescence assay of macrophage polarization *in vitro*

We seeded RAW264.7 cells on 24-well plates (1 × 10^5^ cells/well) and used BSHXD and LPS to process for a certain time. Then we fixed the cells in the pore plate and incubated overnight at 4 °C with anti-CD86 (1:1000, A1199, Abclonal, Wuhan, China) antibody. The Goat Anti-Mouse IgG H&L (Alexa Fluor® 555) (1:1000, ab150114, Abcam) were incubated with cell for 1 h at 37 °C.100 μl Alexa Fluor® 488 Phalloidin (1:20, #8878s, CST, Danvers, USA) added to the cells. The nuclei were stained with DAPI. Fluorescence images were obtained using immunofluorescence microscopy (Thermo Fisher Scientific, Bothell, WA, USA). The THP-1 cells were immunofluorescence stained with the same procedure.

### 2.7 Western blot analysis

RAW264.7 cells (5 × 10^5^ cells/well) were induced with LPS (1 μg/mL) in 6-well plates and treated with BSHXD for a certain time. Total protein was extracted from RAW264.7 using RIPA buffer (P0013C, Beyotime, Shanghai, China) and protease inhibitor cocktail (P1050, Beyotime). Subsequently, 20 μg samples were loaded onto gels and Marker (RM19001, Abclonal) were separated by electrophoresis. The protein bands were transferred from gel to polyvinylidene fluoride membrane and then blocked in blocking solution. The membrane was then incubated with primary antibodies and secondary antibody. Finally, the protein bands were observed with a chemiluminescent HRP substrate (WBKLS0500, Millipore Corporation). The following antibodies were used: IL-1β(#31202, CST), IL-6 (ab259341, Abcam), TNF-α (ab183218, Abcam), CD86 (A1199, Abclonal), AMPKα1 (A1229, Abclonal), *P*-AMPKα1 (#4184, CST), PP2Aα+β (ab168350, Abcam) and β-Actin (#12262,CST), Goat Anti-Mouse (#7074, CST) and Anti-rabbit (#91196, CST).

### 2.8 Flow cytometry

After RAW264.7 cells (5 × 10^5^ cells/well) attached and grew, they were pretreated with BSHXD for 1 h, stimulated with LPS for 24 h, and the RAW264.7 cell suspension was collected and washed three times with PBS. 1 × 10^6^ cells were suspended in 1.5 mL EP tubes and incubated with Fc receptor blocker for 10 min on ice. After 2 washes, PBS containing antibodies were added to the EP tubes. The following antibodies were used: CD86-FITC (#11-0862-82, invitrogen, Carlsbad, USA) and F4/80-APC (#17-4801-82, invitrogen), incubated on ice for 30 min in the dark, and after 2 washes, the cells were suspended in 1 mL of flow cytometry buffer for detection by flow cytometry.

### 2.9 In vivo experiments

All animal experiments were performed in accordance with the Experimental Animal Policy and were approved by the Animal Ethics and Welfare Committee (AEWC) (Approval NO: AEWC-20201005). Mice were on a 12-h light/dark cycle and were given regular food and water. Forty 7-week-old male C57BL/6 J mice were randomly divided into 4 groups (10 mice in each group): control group, vehicle group (injection of LPS), Low-BSHXD group (based on the model group, given daily intragastric administration of BSHXD, daily dose: 13.2225 mg/kg), High-BSHXD group (based on the model group, given daily intragastric administration of BSHXD, daily dose: 26.445 mg/kg). A murine model of inflammatory bone loss stimulated by skull injection of LPS was established [28]. After anesthesia, LPS was injected in the intersection of the sagittal suture with the midpoint of the ears in mice. Starting from the day brfore the operation, an equal amount of water or BSHXD was given by gavage every day, respectively. The high dose amount of BSHXD in mice was calculated based on body surface area [29]. After 2 weeks of drug treatment, mice skull was collected and fixed. In addition, major organs (liver and kidney) were also obtained to assess the systemic toxicity of BSHXD. No major adverse events were recorded in animal experiments (Fig.S3).

### 2.10 Micro-CT evaluation

We randomly selected 3 out of 10 mouse skulls [30]. Skulls were fixed with 4% formaldehyde for at least 24 h and then analyzed using high-resolution micro-CT (SkyScan 1176; SkyScan, Knotich, Belgium). The scan parameters were as following: equidistant resolution: 9 μm, x-ray energy: 50 kV and 500 μA. We used the software provided by the manufacturer to reconstruct the images. For further quantitative analysis, we selected a square area (ROI 3 × 3 × 1 mm) around the LPS injection site. Bone quality parameters, such as bone mineral density (BMD, g/cm^3^), bone volume/tissue volume (BV/TV, %), bone volume (BV, mm^3^) and total volume of pore space (Po.V, mm^3^) were measured on reconstructed images.

### 2.11 Hematoxylin and eosin staining

After micro-CT, the remaining 7 mouse skulls were decalcified in 10% Ethylene Diamine Tetraacetic Acid (EDTA) for 4 weeks, when the skull can be easily penetrated with a fine needle. We trimmed the edges of the skull to preserve the sagittal line of the skull, embedded it in paraffin. To ensure randomness, we randomly selected 3 out of the 7 skull paraffin blocks and prepared coronal slices (5 μm thick) with a microtome. These slices were used as in vivo samples for subsequent histological staining. The slices were stained with hematoxylin and eosin (H&E) and photographed using a Nikon Eclipse Ci-L microscope (Nikon, Tokyo, Japan).

### 2.12 Immunohistochemical analysis

Immunohistochemical staining was used to detect the expression of IL-1β(1:100, #12242, CST),IL-6 (1:50, ab208113, abcam) and TNF-α(1:100, A11534, ABclonal). Skulls sectioned were incubated with the respective primary antibodies overnight at 4 °C. Sections were then washed and incubated with secondary antibody for 30 min at room temperature. Stained sections were all photographed using light microscope. Histomorphological analysis of bones was performed via Panorama Histocytometry Quantitative Analysis System (TissueFAXS Plus, TissueGnostics GmbH, Austria).

### 2.13 Immunofluorescence staining

We also completed immunofluorescence staining (IF) for CD86 (1:200, #19589, CST) and F4/80 (1:200, #70076, CST). After deparaffinization, antigen retrieval and sealing, skull slices were incubated with primary antibody overnight at 4 °C. Then, the slices were washed and incubated with the secondary antibody for binding with the secondary antibody for 60 min. Nuclear staining was performed using DAPI. Finally, the specimen was observed with Nikon Eclipse Ci-L microscope.

### 2.14 Statistical analysis

All experimental data were expressed as mean ± standard deviation (M ± SD). One-way analysis of variance (anova) was used for statistical analysis of more than two groups of data using GraphPad Prism 8.3.0 software. Statistical significance was defined when p < 0.05.

## 3 Results

### 3.1 Identified chemicals in BSHXD

**Fig. 1:**
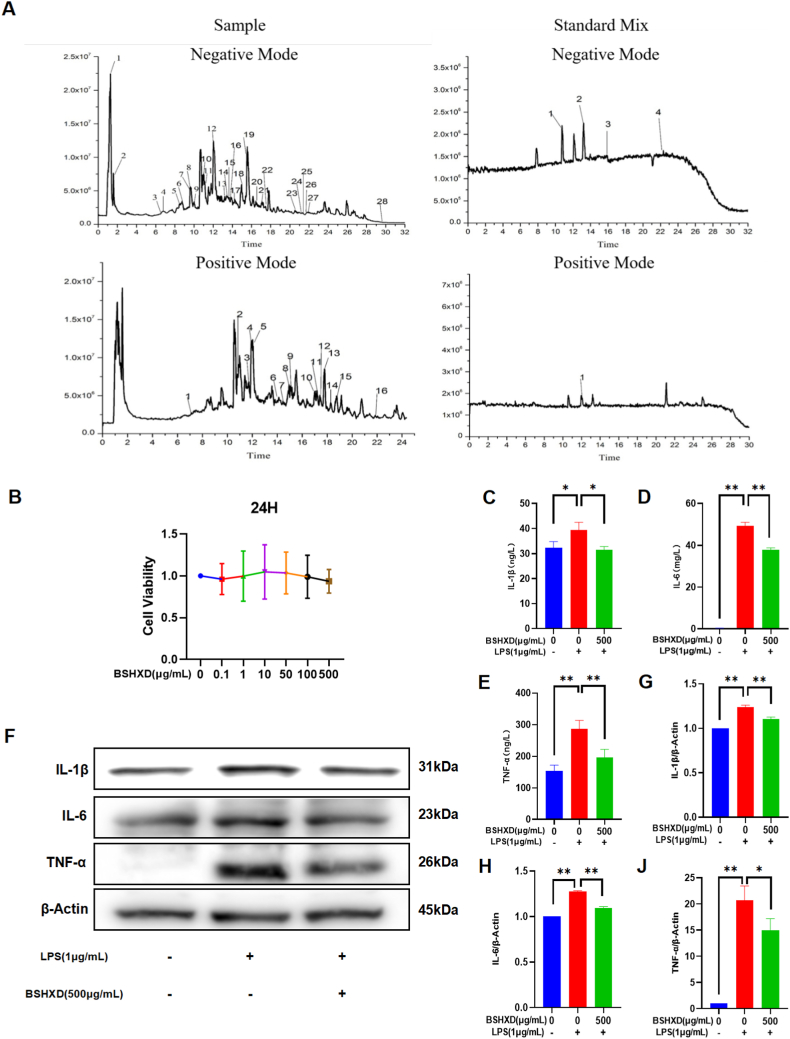
**BSHXD can effectively inhibit the levels of pro-inflammatory cytokines IL-1β, IL-6 and TNF-α.** (A) The total ion chromatography of the standards and the BSHXD mode by UFLC-Q-TOF/MS. (B)cell viability tests. Protein level expressions of inflammatory cytokines IL-1β(C), IL-6 (D)and TNF-α(E) in LPS-treated macrophages (measured by ELISA). (F) IL-1β, IL-6 and TNF-α protein levels, detected by Western blot analysis. (G) IL-1β, (H) IL-6 and (J) TNF-α were normalized to β-Actin and expressed in arbitrary units. Data are presented as mean ± SD of three independent experiments. *p < 0.05, **p < 0.01 compared to LPS group.

After constructing an in-house chemical library of BSHXD, the raw MS data could be compared with the standards and chemical information of the in-house library. The total ion chromatography of the standard mixture and the BSHXD both in positive mode and negative mode were presented in Fig. 1A. The 44 chemicals of the BSHXD could be identified in this study, the detailed information of these chemicals could be seen in the supplementary material (Table S1).

### 3.2 BSHXD reduced LPS-induced release of inflammatory cytokines IL-1β, IL-6, TNF-α in RAW264.7

We performed a cytotoxicity assay (CCK-8 assay) to rule out the possibility of BSHXD toxicity on RAW264.7 cells. The results show that BSHXD concentration in the range of 0–500 μg/mL has no toxicity. (Fig. 1B). Therefore, in the subsequent experiments, we chose the concentration of 500 μg/mL for in vitro evaluation.

ELISA results showed that the expression levels of IL-1β(Fig. 1C), IL-6 (Fig. 1D) and TNF-α(Fig. 1E) in the supernatant of RAW264.7 cells were significantly increased after LPS stimulation (39.42 ± 3.076 ng/L, 49.24 ± 1.766 mg/L and 286.3 ± 27.12 ng/L, p < 0.05). The application of BSHXD significantly lower the expression levels of IL-1β, IL-6 and TNF-α in the supernatant (31.55 ± 1.296 ng/L, 37.94 ± 0.8869 mg/L, and 196.4 ± 25.25 ng/L, p < 0.05). The same result was manifested in WB (Fig. 1F–J). The above results indicate that BSHXD can effectively inhibit the secretion of pro-inflammatory cytokines and reduce the inflammatory response caused by LPS.

### 3.3 BSHXD inhibited LPS-induced M1 polarization of RAW264.7 macrophages

**Fig. 2:**
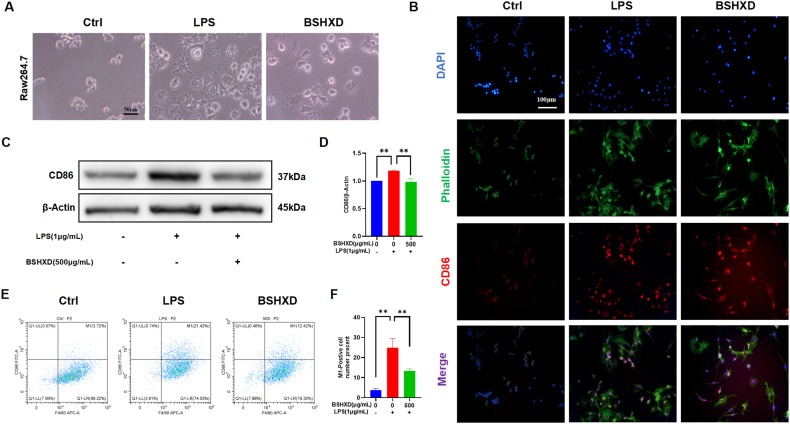
**BSHXD inhibited LPS-induced M1 polarization of RAW264.7 macrophages.** (A) The morphology of RAW264.7 cells observed by light microscope. (B) Immunofluorescence staining of RAW264.7 cells. (C)The protein level of CD86, detected by Western blot analysis. (D) CD86 was normalized to β-Actin and expressed in arbitrary units. (E) The proportion of F4/80+ and CD86^+^ in RAW264.7 cells, detected by flow cytometry. N = 3, data are expressed as mean ± SD. *p < 0.05, **p < 0.01 compared to LPS group.

M1 macrophages can secrete inflammatory factors such as IL-1β, IL-6, TNF-α [13]. To further examine the effect of BSHXD on the M1 polarization of RAW246.7 cells, RAW246.7 cells were intervened with BSHXD for 24 h. The morphological changes of RAW246.7 in each group (Fig. 2A) showed that after stimulation with LPS, elongated pseudopodia appeared on RAW264.7 cells, indicating LPS induced M1 polarization. The pseudopodia ratio decreased significantly after BSHXD treatment. The same results were also observed in THP-1 cells (Fig. S1A&B).

CD86 is an important marker of M1 polarization in macrophages, and we performed immunofluorescence staining, WB and flow cytometry to assess the changes in expression of CD86. The results of immunofluorescence staining showed (Fig. 2B) that the CD86 expression was significantly increased after stimulation of LPS, while this increase was inhibited by BSHXD. The WB results (Fig. 2C&D) were consistent with immunofluorescence staining and the difference is statistically significant. These results indicated that LPS induce the M1 polarization of macrophage while this effect can be counteracted by BSHXD. We further determined the proportion of M1-type macrophages by flow cytometry (Fig. 2E&F). The proportion of M1-type macrophages in RAW264.7 cell was significantly increased in LPS group and decreased after BSHXD treatment (13.36% ± 0.9829% VS 24.80% ± 4.619%, p < 0.05). These results suggest that BSHXD can inhibit the polarization of macrophages towards M1 type.

### 3.4 BSHXD inhibited M1 polarization of macrophage by activating AMPK signaling pathway

**Fig. 3:**
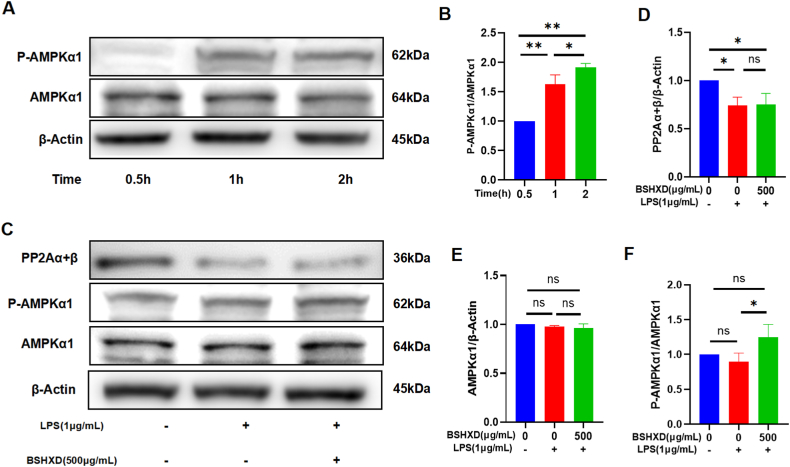
**Activation of AMPK signaling pathway by BSHXD.** (A) *P*-AMPKα1 and AMPKα1 protein levels at various times, detected by Western blot analysis. (B) The relative levels of *P*- AMPKα1/AMPKα1. (C) PP2Aα+β, *p*-AMPKα1 and AMPKα1 protein levels, detected by Western blot analysis. The relative levels of (D) PP2Aα+β/β-Actin, (E)AMPKα1/β-Actin and (F) *P*- AMPKα1/AMPKα1. N = 3. Data are presented as mean ± SD of three independent experiments. *p < 0.05, **p < 0.01 compared to LPS group.

AMPK pathway is an important pathway regulating macrophage polarization. We further studied the effect of BSHXD on the AMPK pathway in RAW264.7 cells (Fig. 3A&B). The results showed that the expression of *p*-AMPKα1 gradually increased at 0.5 h, 1 h, 2 h while the expression of AMPKα1 was unchanged. Subsequently, we performed Western blot analysis to evaluate the effect of BSHXD on *p*-AMPKα1, AMPKα1 and PP2Aα+β (Fig. 3C–F). The level of *p*-AMPKα1 decreased after 2 h treatment of LPS, while BSHXD restored *p*-AMPKα1 activity. Next, we investigated the effect of BSHXD on PP2Aα+β. There was no significant change in PP2Aα+β expression level after LPS treatment for 2 h. These results suggest that BSHXD increased *p*-AMPKα1 expression, but not PP2Aα+β.

**Fig. 4:**
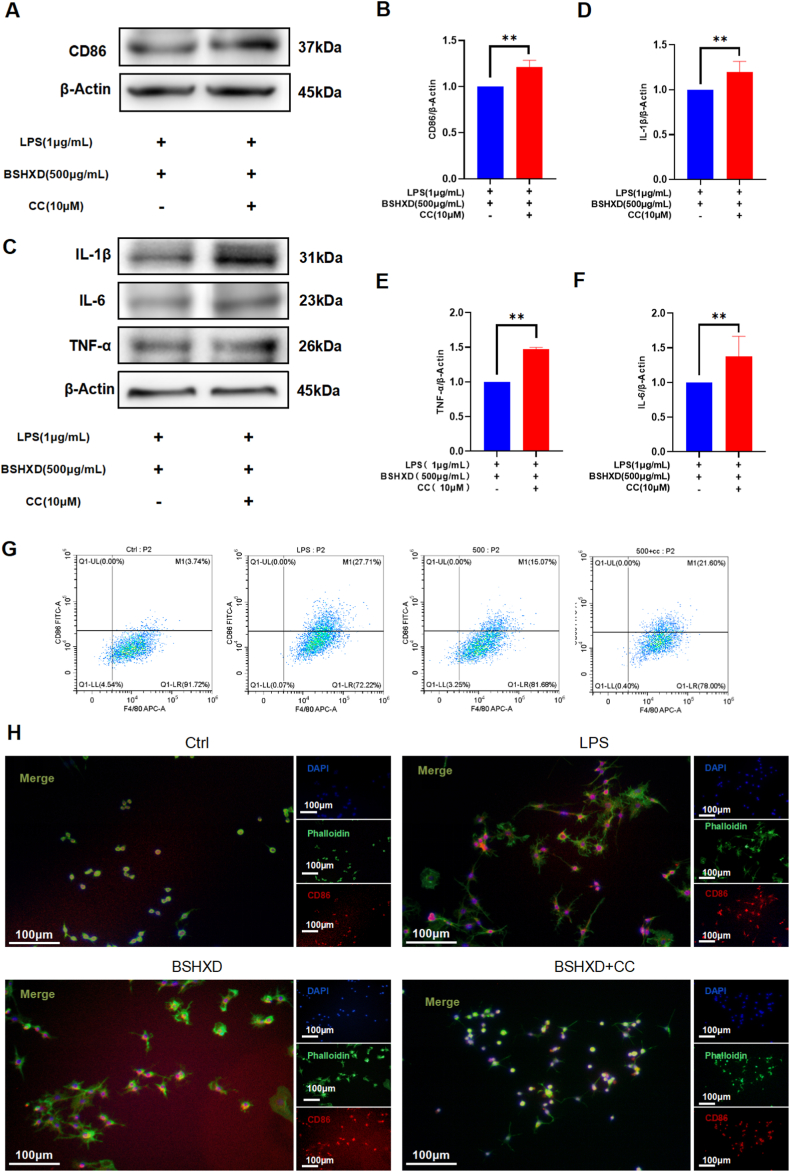
**BSHXD inhibits inflammatory factors and M1 macrophages through activating AMPK.** (A) The CD86 protein level, detected by Western blot analysis. (B) CD86 was normalized to β-Actin and expressed in arbitrary units. (C) IL-1β, IL-6 and TNF-α protein levels, detected by Western blot analysis. (D) IL-1β, (E) IL-6 and (F) TNF-α were normalized to β-Actin and expressed in arbitrary units. (G) The proportion of F4/80^+^ and CD86^+^ in RAW264.7 cells, detected by flow cytometry. (H) Typical images of immunofluorescence staining: red (CD86), green (F4/80), and blue (DAPI). Scale bar indicates 100 μm.N = 3. Data are presented as mean ± SD of three independent experiments. *p < 0.05, **p < 0.01.

To further verify the role of AMPK pathway in the anti-inflammatory mechanism of BSHXD, we used the AMPK inhibitor Dorsomorphin (Compound C, S7306, Selleck, Shanghai, China) to downregulate AMPK activity. The results of WB showed that the inhibition of CD86 (Fig. 4A&B) and inflammatory factors IL-1β, IL-6 and TNF-α (Fig. 4C–F) by BSHXD were both attenuated, when the AMPK pathway is inhibited. The results of flow cytometry (Fig. 4G) showed that after LPS stimulation, the proportion of M1 macrophages was increased, while BSHXD intervention was decreased. Compared with BSHXD intervention, the proportion of M1 macrophages increased slightly after inhibition of ampk pathway, but still lower than that of LPS stimulated group. The result of immunofluorescence (Fig. 4H) were consistent with Flow cytometry. The results showed that the inhibition of BSHXD on pro-inflammatory cytokines and M1 macrophages was partially weakened, when the AMPK pathway is inhibited. Additionally, these changes are not related to Compound C (Fig. S2).

### 3.5 BSHXD inhibited LPS-induced inflammatory bone loss

**Fig. 5:**
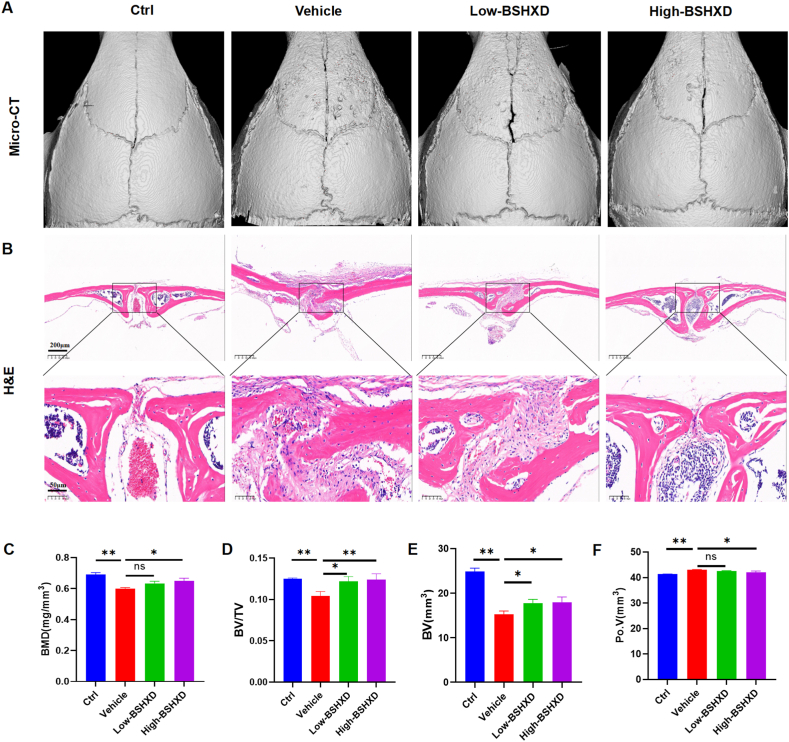
**BSHXD inhibited LPS-induced inflammatory bone loss.** (A) Micro-CT image of mouse skulls. (B) H&E staining of the skulls. Scale bar indicates 200 and 50 μm. Computations for (C)BMD, (D)BV/TV, (E)BV and (F) Po.V. N = 3. All data were expressed as the mean ± SD. ns, no significance, *p < 0.05, **p < 0.01, compared with the vehicle group.

After collecting the skull of each group of mice, Micro-CT examination was performed (Fig. 5A). The results showed that the skull of the mice in the LPS group showed obvious osteopenia and disorganized trabecular bone, while the skull of the blank group was intact. Bone loss on the skull surface was significantly reduced after treatment with BSHXD, suggesting that BSHXD protects bone from inflammation and preserves bone integrity. Quantitative analysis showed that compared with the blank group (Fig. 5C–F), the BMD, BV/TV and BV of the LPS group decreased by 12.95%, 16.35% and 38.70% respectively (p < 0.05), and Po.V of the LPS group increased by 4.07% (p < 0.05). On the other hand, BMD, BV/TV and BV were significantly up regulated after BSHXD treatment, and Po.V decreased significantly. The bone quality parameters such as BMD, BV/TV and BV in the LPS group were both significantly decreased, while those in the BSHXD intervention group were significantly increased, but the results of Po.V were reversed. (BMD mg/mm^3^: 0.6476 ± 0.0195 vs 0.6335 ± 0.0132 vs 0.6005 ± 0.0059, BV/TV: 0.1238 ± 0.0072 vs 0.1217 ± 0.0059 vs 0.1043 ± 0.0054, BV mm^3^: 17.97 ± 1.181 vs 17.75 ± 0.8600 vs 15.22 ± 0.7839,Po.V mm^3^: 41.96 ± 0.5670 vs 42.57 ± 0.1053 vs 42.99 ± 0.1430, respectively). The results of H&E staining also showed (Fig. 5B) that the LPS group showed obvious inflammatory infiltration on the skull surface while the inflammatory infiltration was alleviated after BSHXD treatment.

**Fig. 6:**
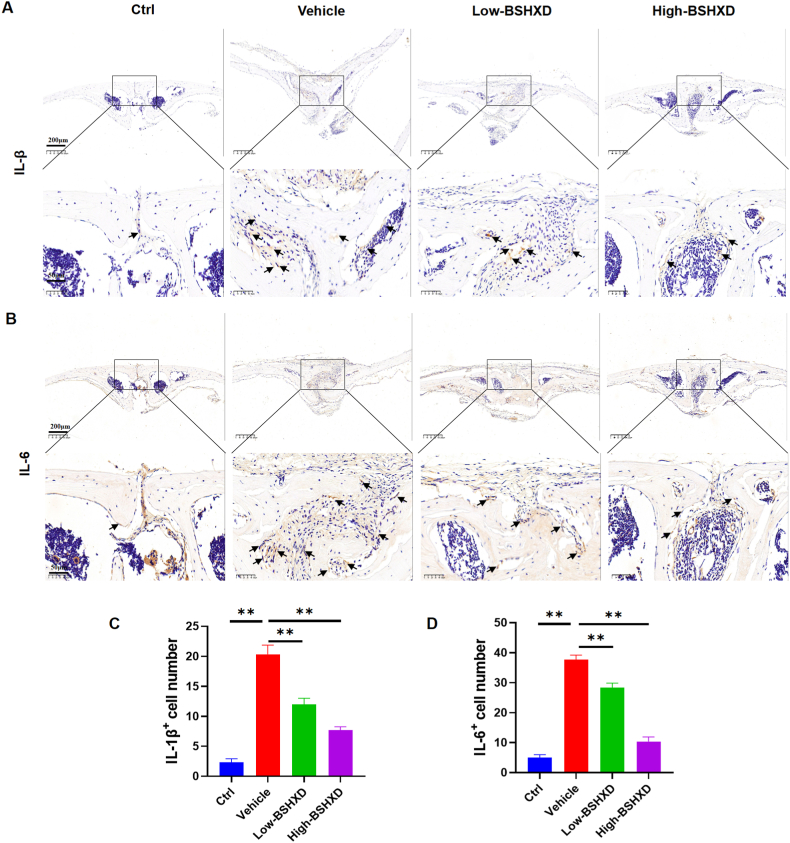
**BSHXD inhibited LPS-induced inflammatory bone loss.** Immunohistochemical staining of inflammatory factors in mouse skull: (A) IL-1β and (B) IL-6. Number of positive cells: (C) IL-1β and (D)IL-6. Scale bar indicates 200 and 50 μm. N = 3. All data were expressed as the mean ± SD. *p < 0.05, **p < 0.01, compared with the vehicle group.

**Fig. 7:**
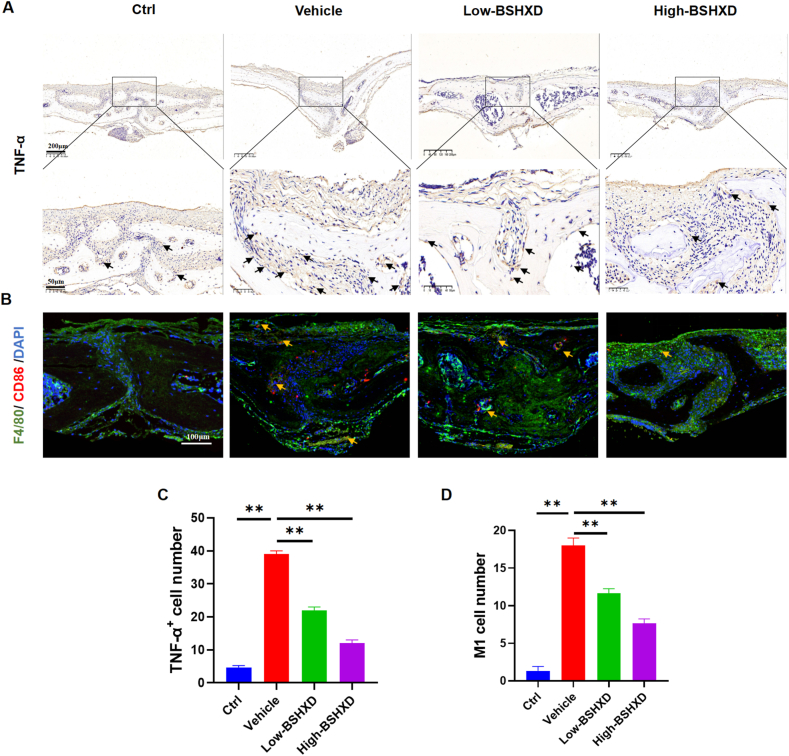
**BSHXD inhibited LPS-induced inflammatory bone loss.** (A)Immunohistochemical staining of TNF-α in mouse skull. Scale bar indicates 200 and 50 μm. (B)Immunofluorescence staining in mouse skull: red (CD86), green (F4/80), and blue (DAPI). Scale bar indicates 100 μm. (C) Number of TNF-α positive cells. (D) Number of CD86 positive cells. N = 3. All data were expressed as the mean ± SD. *p < 0.05, **p < 0.01, compared with the vehicle group.

Immunohistochemical staining showed (Fig. 6A–D, Fig. 7A&C) that compared with blank group, LPS group showed increased levels of IL-1β, TNF-α, and IL-6, which mainly distributed in the inflammatory cells around the erosion area. On the contrary, few positive staining of these inflammatory cytokines was seen after BSHXD treatment. CD86 immunofluorescence staining showed (Fig. 7B&D) that a large number of M1 phenotype macrophages (CD86^+^, red) were distributed in the inflammatory area of LPS group. In the BSHXD group, however, the increase in CD86^+^ M1 macrophages was reversed. These consistent results from Micro-CT, H&E staining, immunohistochemical staining and immunofluorescence staining further indicated that BSHXD can inhibit the M1 polarization of macrophages, thereby alleviating local inflammation and reducing bone loss.

## 4 Discussion

**Scheme 1:**
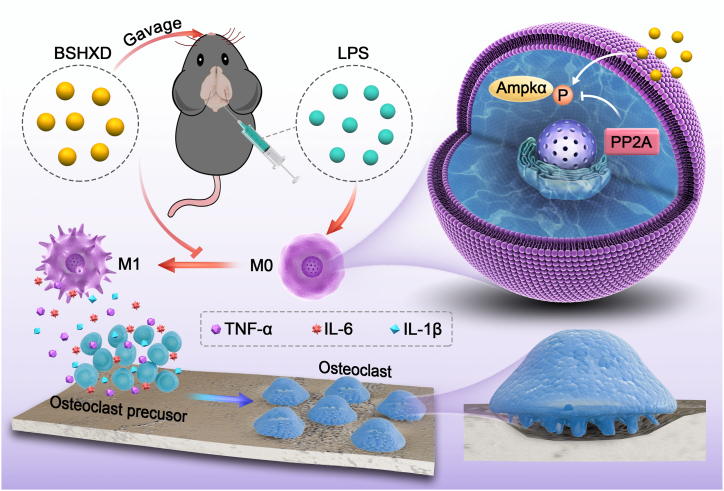
BushenHuoxue Decoction suppresses M1 Macrophage Polarization and inflammation, prevents LPS Induced Bone Loss, by Activating AMPK Pathway.

In the present study, we confirmed the anti-inflammatory effect of BSHXD and explored its underlying mechanism. BSHXD has a therapeutic effect on inflammatory bone loss. It alleviates skull bone loss and inhibits the level of proinflammatory cytokines and the expression of M1 macrophages. As shown in Scheme 1, the main findings of our study include: (1) BSHXD treatment attenuated LPS-induced local inflammatory bone loss in mice. (2) The inhibitory effect of BSHXD was associated with the inhibition of M1 polarization in RAW264.7 macrophages by promoting AMPK phosphorylation.

Previous research data showed that traditional Chinese medicine can inhibit inflammation and reduce bone loss. Its mechanism is related to inhibition of M1 polarization of macrophages and down-regulation of proinflammatory cytokines [21,[31], [32], [33], [34], [35]]. Substantial evidence suggests that macrophages are highly plastic to the skeletal inflammatory microenvironment [14,36], and that macrophages including proinflammatory M1 macrophages and repaired M2 macrophages, function as immune response regulators [37]. M1 polarization of macrophage plays an important role in the onset and progression of inflammatory bone loss [38,39]. Previous research showed that curcumin suppressed the percentage of M1 macrophages, while inhibiting the release of pro-inflammatory cytokines TNF-α and IL-6 [40]. Isobavachalcone can inhibit macrophage M1 polarization and reduce osteoporosis in ovariectomized mice [21]. It was also observed in our study that M1 macrophages were significantly increased in LPS-induced inflammatory bone loss, which is consistent with literature reports [31]. The proportion of M1 macrophages significantly decreased after BSHXD administration and inflammatory bone loss was reduced. At the same time, in vitro experiments also confirmed that BSHXD can reduce the proportion of M1 macrophages and inhibit inflammation. It was confirmed that suppressing inflammatory microenvironment and inhibiting M1 macrophage polarization play a key role in the BSHXD action of preventing and treating inflammatory bone loss.

AMPK is a major cellular energy sensor that regulates metabolism in eukaryotes, and an increasing number of studies have shown that AMPK activation exerts anti-inflammatory effects in a variety of immune cells, including macrophages, neutrophils, T cells, and mast cells. We observed that *p*-AMPK was inhibited after LPS stimulation, which is consistent with the previous literature [41]. Also, our study showed that BSHXD was able to upregulate AMPK levels and affected the phosphorylation of AMPKα1. Interestingly, we found that this regulation was completed independent of PP2Aα+β. Furthermore, the addition of AMPK inhibitor compound C (polymorphine) reversed the inhibitory effect of BSHXD on M1 polarization and expression of pro-inflammatory cytokines in macrophages, further confirming that the mechanism of BSHXD action is closely related to the activation of AMPK. Collectively, our results suggested that the effect of BSHXD on inhibiting M1 polarization of macrophages and reducing inflammation level is achieved by activating AMPK.

The current study had several limitations. Firstly, BSHXD is a traditional Chinese medicine compound composed of 9 kinds of herbs. Therefore, BSHXD is very complex. However, the exact active ingredients of BSHXD still need to be further studied. Secondly, our study found that BSHXD can reduce inflammatory bone loss. Then we used ampk inhibitors in cell experiments, but there was no relevant in vivo experiment. In future research, we need to use gene knockout mice to confirm these results. Finally, BSHXD are mixtures of various Chinese medicines with multiple targets and mechanisms of action. The effect of BSHXD on bone loss may also be related to other pathways or targets, which needs further study. In addition, in inflammatory bone loss, there is also an imbalance of osteoblast/osteoclast activity, which is crucial to effectively alleviate bone loss. However, the mechanism of BSHXD on osteoblasts and osteoclasts is still unclear, which will be our future research content.

## 5 Conclusion

Our study found that BSHXD successfully attenuated inflammatory bone loss via inhibiting M1 macrophage polarization and reducing inflammatory activity (Scheme 1). Based on these results, BSHXD is expected to be a promising therapeutic strategy for the treatment of inflammatory bone loss and other inflammatory diseases.

## Funding

This work was supported by the National Natural Science Foundation of China (82074473, and 82104892), the 10.13039/501100004608Natural Science Foundation of Jiangsu Province (BK20191201 and BE2020666), Jiangsu Province "333 Project" research project (BRA2020129), the Elderly Health Research Project of Jiangsu Province (LK2021043, and LR2021024), Suzhou Special Project for Diagnosis and Treatment of Clinical Key Diseases (LCZX202221), Key Disciplines in Suzhou (SZXK202120), Suzhou Science and Technology Development Plan Project (SYSD2017008, SYSD2019007, SYSD2020009, SYSD2021175 SKY2021001, SKY2021014, SKY2021015, SKJY2021002 and SKJYD2021175), the Suzhou Health Personnel Training Project (GSWS2019074 and GSWS2020103), the Suzhou Health System Youth Science and Technology Project (KJXW2021066, KJXW2022060 and KJXW2022063). The Zhangjiagang Health Personnel Training Project (ZJGWSRC2020002), the Zhangjiagang Health System Youth Science and Technology Project (ZJGQNKJ202010, ZJGQNKJ202031, ZJGQNKJ202110, ZJGQNKJ202133, ZKS2007, ZKS2121, ZKS2126, ZKS2129, ZKS2030 and ZKS2032).

## Author contribution statement

Shuangshuang Chen, Feng Zhu, Zhifang Wang, Long Xiao, Dechun Geng and Zhirong Wang: Conceived and designed the experiments.

Shuangshuang Chen, Feng Zhu, Qi Zhuang, Yajun Li, Yunshang Yang and Chengcheng Feng,: Performed the experiments.

Shuangshuang Chen, Zhirong Wang, Lihong Tao, Haiwei Shi, Like Zhu, Long Xiao, Dechun Geng and Zhifang Wang: Analyzed and interpreted the data.

Lihong Tao, Jiandong Shi, Long Xiao and Zhifang Wang: Contributed reagents, materials, analysis tools or data.

Shuangshuang Chen, Lihong Tao, Long Xiao, and Dechun Geng: Wrote the paper.

## Data availability statement

Data will be made available on request.

## Additional information

Supplementary content related to this article has been published online at [URL].

## Declaration of competing interest

The authors declare that the study was conducted in the absence of any business or financial relationships that could be construed as potential conflicts of interest.
