# Supplementary material for: BushenHuoxue decoction suppresses M1 macrophage polarization and prevents LPS induced inflammatory bone loss by activating AMPK pathway
Source: Heliyon. 2023 Apr 19;9(5):e15583. doi: 10.1016/j.heliyon.2023.e15583 (PMC10160506; doi:10.1016/j.heliyon.2023.e15583)
Supplement: Multimedia component 1 [file mmc1.docx]

**
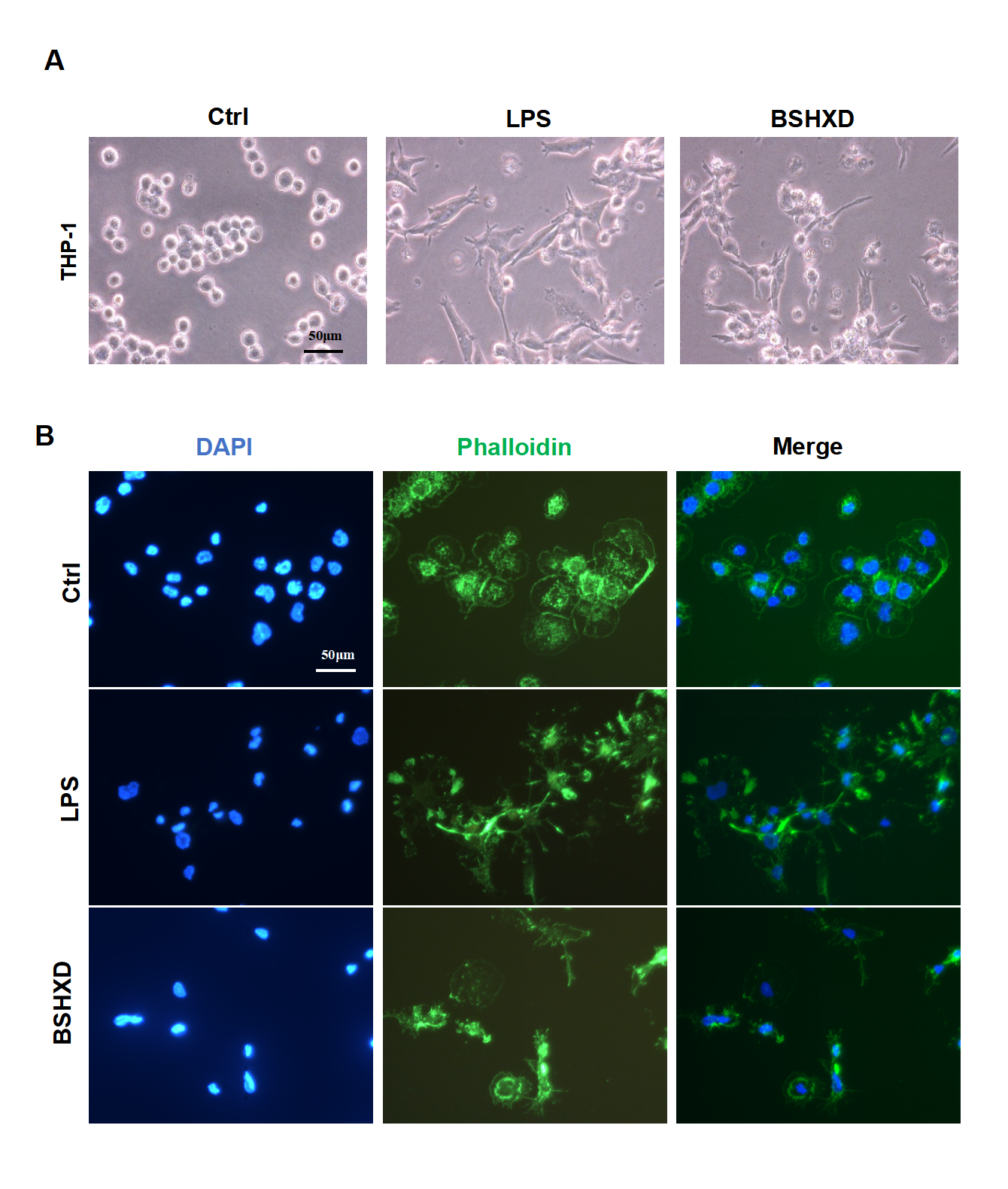
**

**Figure S1. BSHXD inhibited LPS-induced M1 polarization of THP-1 macrophages.** (A)The morphology of THP-1 cells observed by light microscope. (B) Representative immunofluorescent staining images of THP-1 cells: red (M1 marker: CD86), green (M0 marker: F4/80), and blue (DAPI, directing against nuclei). n=3. data are expressed as mean±SD. *p<0.05, **p<0.01 compared to LPS group.


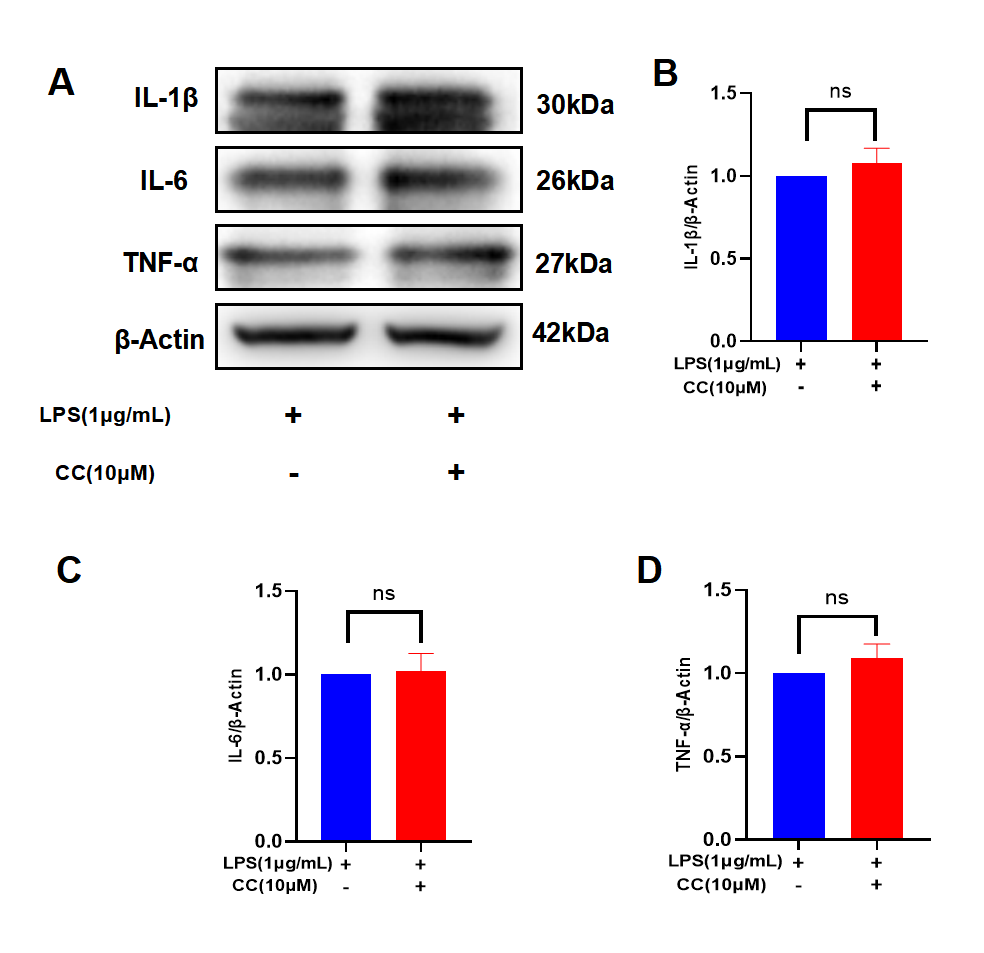


**Figure S2.** **Compound C did not suppress LPS-induced activation of inflammation in vitro.** (A)inflammatory cytokines IL-1β, IL-6, TNF-α，detected by western blot analysis. (B) The relative levels of IL-1β/β-Action(B), IL-6/β-Action(C), TNF-α/β-Action(D). n=3. All data were expressed as the mean ± SD, ns. no significance.


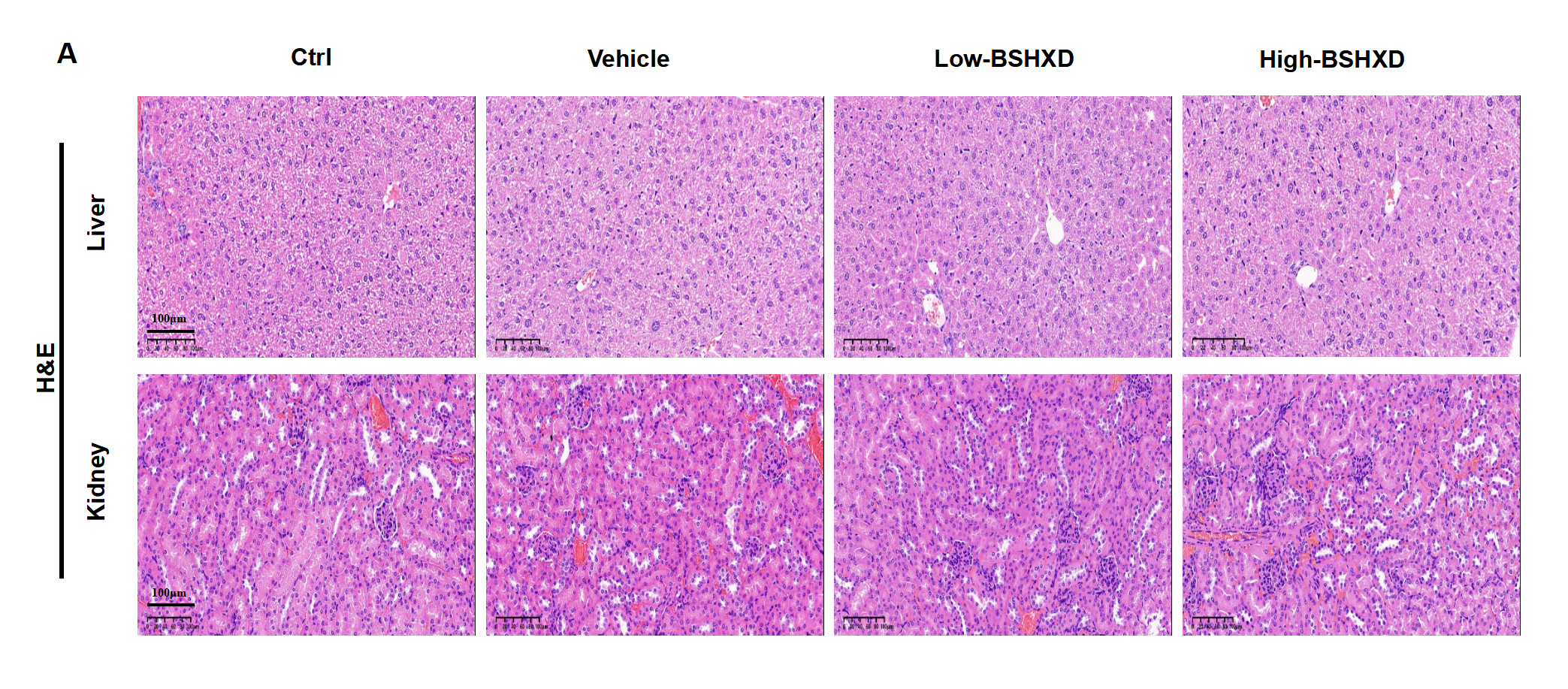


**Figure S3. BSHXD has no toxicity for the mice in vivo.** H&E staining of (A) liver and (B) kidney.

**Table S1. Characterization of the main chemical constituents in BSHXD by UFLC–Q-TOF-MS/MS**

| **ID** | **Retention Time/min** | **Scan Model** | **MS^1^** | **ppm** | **MS^2^** | **Adduct** | **Formula** | **Identification** |
| --- | --- | --- | --- | --- | --- | --- | --- | --- |
| N 1 | 1.42 | Negative | 191.0203 | 5.76 | 173.0389 129.0411 111.0276 | -H | C_6_H_8_O_7_ | Citric acid |
| N 2 | 1.71 | Negative | 361.114 | 1.38 | 199.0969 169.0418 | -H | C_15_H_22_O_10_ | Catalpol |
| N 3 | 6.61 | Negative | 197.0451 | 4.57 | 179.0650 135.0677 | -H | C_9_H_10_O_5_ | Danshensu |
| N 4 | 6.9 | Negative | 179.357 | 5.58 | 135.068 107.0681 | -H | C_9_H_8_O_4_ | Caffeic acid |
| N 5 | 9.18 | Negative | 495.1521 | 0.40 | 495.2348 137.0473 93.0502 | -H | C_23_H_28_O_12_ | Oxypaeoniflorin |
| N 6 | 9.3 | Negative | 387.1297 | 1.55 | 387.1578 225.1144 | -H | C_17_H_24_O_10_ | Geniposide |
| N 7 | 9.54 | Negative | 169.0148 | 2.96 | 125.0462 124.0283 97.0464 69.0478 | -H | C_7_H_6_O_5_ | Gallic acid |
| N 8 | 9.78 | Negative | 345.152 | 8.40 | 183.1343 179.0854 165.1147 | -H | C_16_H_26_O_8_ | Rehmapicroside |
| N 9 | 10.23 | Negative | 301.0359 | 1.73 | 301.0873 179.0294 151.02952 | -H | C_15_H_10_O_7_ | Quercetin |
| N 10 | 11.36 | Negative | 785.2566 | 7.90 | 623.3254 161.0514 | -H | C_35_H_46_O_20_ | Echinacoside |
| N 11 | 11.99 | Negative | 479.1532 | 5.63 | 479.1646 327.1650 121.0506 77.0538 | -H | C_23_H_28_O_11_ | Paeoniflorin |
| N 12 | 12.87 | Negative | 491.1195 | 2.24 | 283.1097 268.0833 211.0749 239.0772 | +HCOO^-^ | C_22_H_22_O_10_ | Calycosin-7-glucoside |
| N 13 | 13.26 | Negative | 313.0724 | 1.92 | 269.1822 109.0469 | -H | C_17_H_14_O_6_ | Salvianolic F |
| N 14 | 13.32 | Negative | 623.1987 | 1.77 | 461.2439 161.0518 | -H | C_29_H_36_O_15_ | VERPROSIDE |
| N 15 | 13.32 | Negative | 525.3051 | 1.35 | 479.3845 319.2456 159.1295 | +HCOO^-^ | C_27_H_44_O_7_ | Lnokosterone |
| N 16 | 13.44 | Negative | 813.2856 | 4.80 | 637.3364 175.0692 | -H | C_37_H_50_O_20_ | Jioglutoside |
| N 17 | 14.54 | Negative | 525.306 | 0.36 | 479.2380 159.0710 319.1349 | +HCOO^-^ | C_27_H_44_O_7_ | β-Ecdysone |
| N 18 | 14.94 | Negative | 793.4349 | 3.88 | 673.3429 631.3114 | -H | C_42_H_66_O_14_ | Zingibroside R1 |
| N 19 | 15.59 | Negative | 299.0557 | -1.37 | 299.1025 284.0813 239.0768 | -H | C_16_H_12_O_6_ | pratensein |
| N 20 | 16.51 | Negative | 283.0619 | 2.47 | 268.0835 211.0748 239.0739 195.0779 | -H | C_16_H_12_O_5_ | Calycosin |
| N 21 | 16.68 | Negative | 475.1222 | -2.72 | 267.1101 252.0862 | +HCOO^-^ | C_22_H_22_O_9_ | Ononin |
| N 22 | 16.88 | Negative | 651.2346 | 6.76 | 475.2606 193.0838 175.0675 | -H | C_31_H_40_O_15_ | Martynoside |
| N 23 | 20.66 | Negative | 717.1469 | 0.28 | 519.1791 321.0932 | -H | C_36_H_30_O_16_ | Salvianolic acid B |
| N 24 | 21.13 | Negative | 267.0668 | 1.95 | 252.0844 195.0777 223.0777 132.0449 | -H | C_16_H_12_O_4_ | formononetin |
| N 25 | 21.31 | Negative | 953.4388 | 0.03 | 909.5980 851.5877 793.5697 | -H | C_47_H_70_O_20_ | Achyranthoside D |
| N 26 | 21.35 | Negative | 829.4527 | -6.40 | 783.5804 829.5993 | +HCOO^-^ | C_41_H_68_O_14_ | Astragaloside A |
| N 27 | 21.43 | Negative | 955.4959 | 5.34 | 835.5969 793.5932 | -H | C_48_H_76_O_19_ | Ginsenoside Ro |
| N28 | 29.96 | Negative | 117.0196 | 2.56 | 100.9433 | -H | C_4_H_6_O_4_ | Succinic acid |
| P1 | 7.19 | Positive | 501.5028 | 0.60 | 501.1987 369.1521 | +H | C_26_H_28_O_10_ | Icariin-7-o-xyloside |
| P 2 | 11.61 | Positive | 203.1956 | 4.92 | 187.0734 | +H | C_11_H_6_O_4_ | xanthotoxol |
| P 3 | 12.03 | Positive | 449.1099 | 4.68 | 449.1952 303.1069 | +H | C_21_H_20_O_11_ | Quercitrin |
| P 4 | 12.24 | Positive | 433.1142 | 5.54 | 433.1941 415.1803 397.1678 | +H | C_21_H_20_O_10_ | 3-Hydroxypuerarin |
| P 5 | 12.31 | Positive | 285.0759 | 0.70 | 285.1265 270.1008 | +H | C_16_H_12_O_5_ | Wogonin |
| P 6 | 14.68 | Positive | 825.7876 | 4.72 | 517.2697 355.1844 | +H | C_38_H_48_O_20_ | Hydro-Epimedin C |
| P 7 | 14.85 | Positive | 795.7533 | 5.53 | 517.2673 355.1841 | +H | C_37_H_46_O_19_ | Icariin E |
| P 8 | 15.22 | Positive | 663.6471 | 6.03 | 355.1850 299.1124 | +H | C_32_H_38_O_15_ | DemethylSagittatoside A |
| P 9 | 15.56 | Positive | 231.0756 | 5.63 | 231.0693 187.0641 147.0722 | +H | C_14_H_14_O_3_ | osthenol |
| P 10 | 16.92 | Positive | 677.6679 | 2.51 | 531.2874 369.2030 313.1295 | +H | C_33_H_40_O_15_ | Icariin |
| P 11 | 16.99 | Positive | 839.8124 | 2.62 | 531.187 369.2016 313.1278 | +H | C_39_H_50_O_20_ | Epimedin A |
| P 12 | 17.25 | Positive | 809.7813 | 3.70 | 531.2865 369.2026 313.1296 | +H | C_38_H_48_O_19_ | Epimedin B |
| P 13 | 18.28 | Positive | 821.792 | 3.65 | 531.2869 369.2028 313.1297 | +H | C_39_H_48_O_19_ | 1.3-isoprene Epimedin C |
| P 14 | 18.29 | Positive | 881.8439 | 3.40 | 719.3833 531.2857 369.2015 313.1296 | +H | C_41_H_52_O_21_ | Epimedin I |
| P 15 | 18.52 | Positive | 837.7978 | 4.06 | 531.2895 369.2004 313.1308 | +H | C_39_H_48_O_20_ | 1.3-isoprene Epimedin A |
| P 16 | 22.19 | Positive | 803.778 | 2.12 | 369.2017 313.1300 | +H | C_39_H_46_O_18_ | 3-O - (acetoxy) rhamnose-2-o - (m-diacetoxy) glucose Icariin |
