# Supplementary material for: BushenHuoxue decoction suppresses M1 macrophage polarization and prevents LPS induced inflammatory bone loss by activating AMPK pathway
Source: Heliyon. 2023 Apr 19;9(5):e15583. doi: 10.1016/j.heliyon.2023.e15583 (PMC10160506; doi:10.1016/j.heliyon.2023.e15583)

Western Blot original images of Raw 264.7 cells

Fig.1.F

IL-1β 31kDa

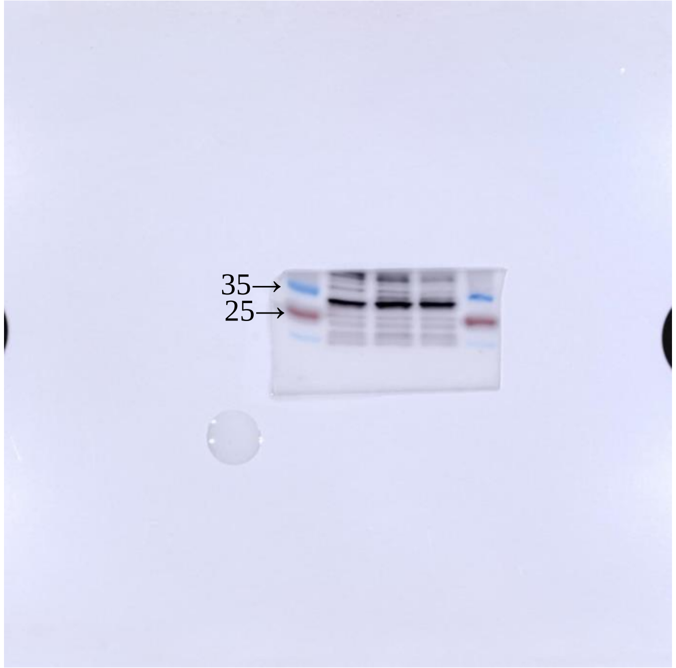

IL-6 23kDa

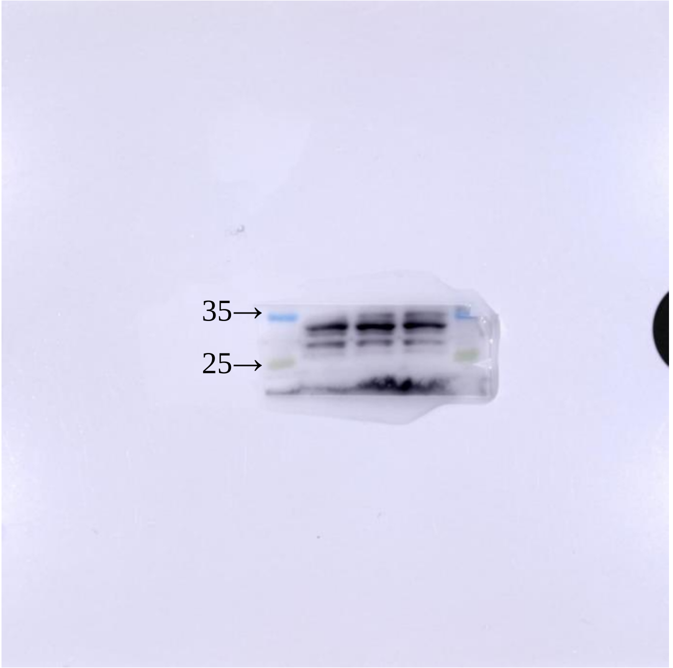

Fig.1.F

TNF-α 26kDa

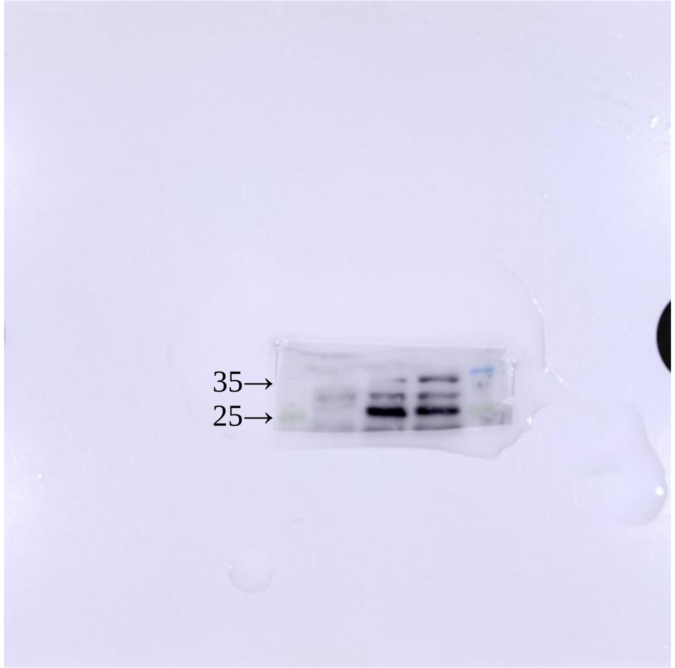

β-Actin 45kDa

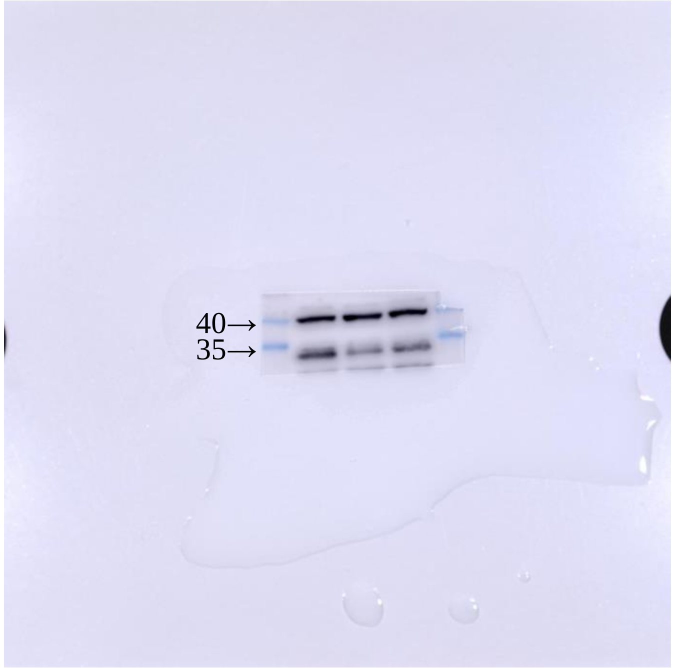

Fig.2.C

CD86 37kDa

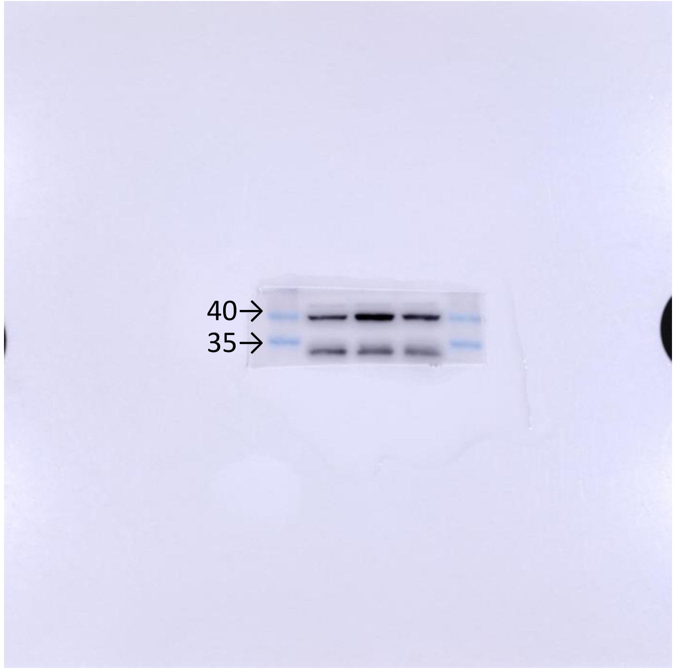

β-Actin 45kDa

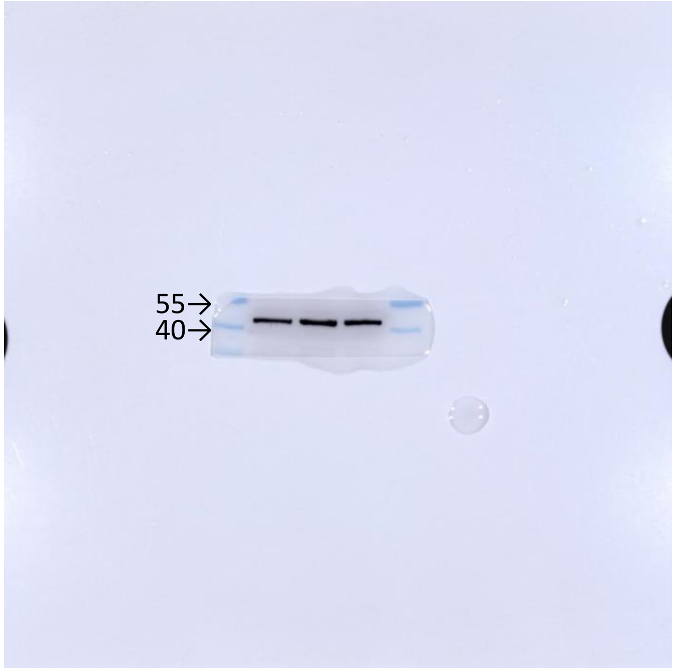

Fig.3.A

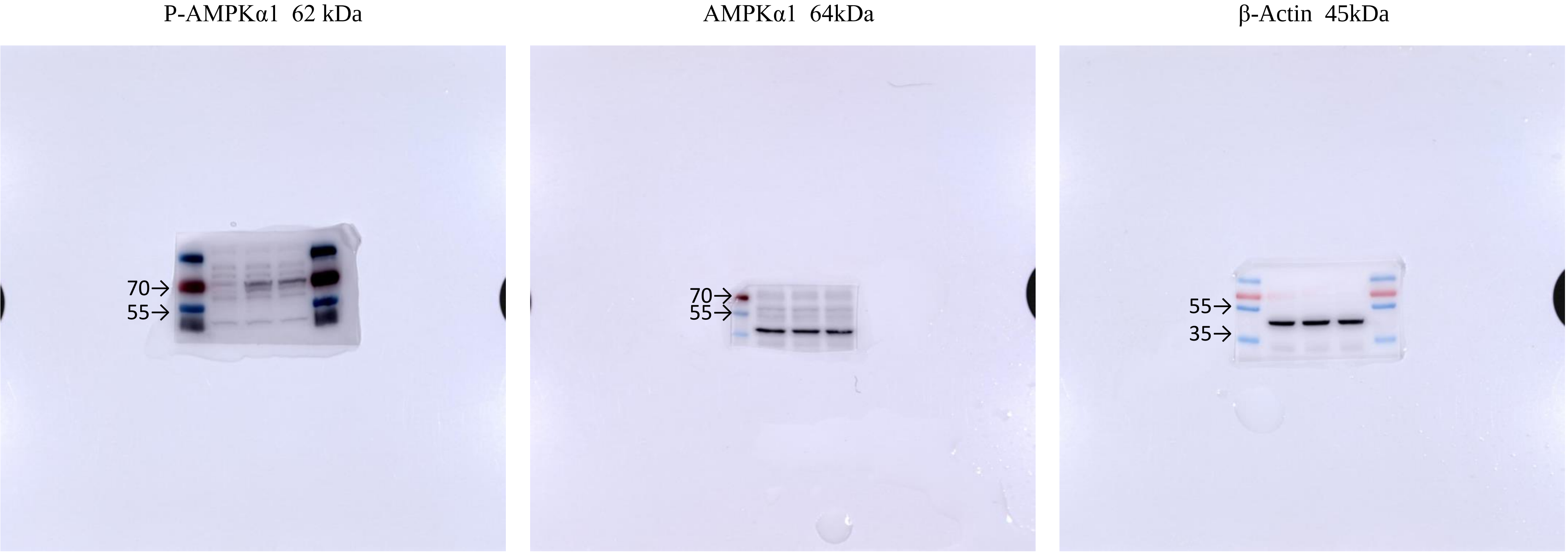

Fig.3.C

PP2Aα+β 36kDa

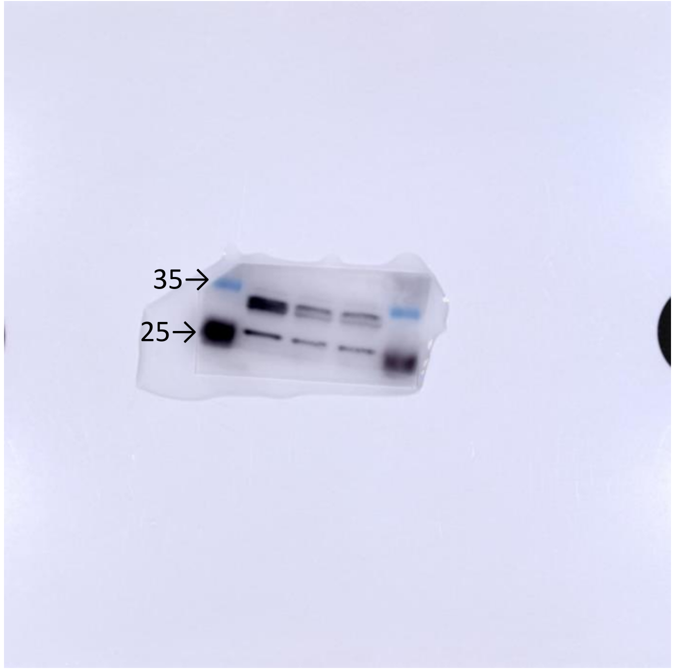

P-AMPKα1 62kDa

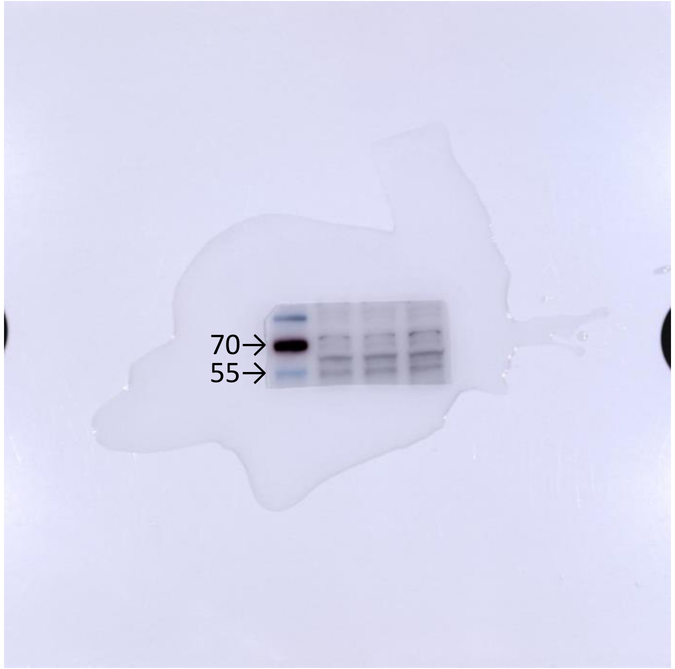

Fig.3.C

AMPKα1 64kDa

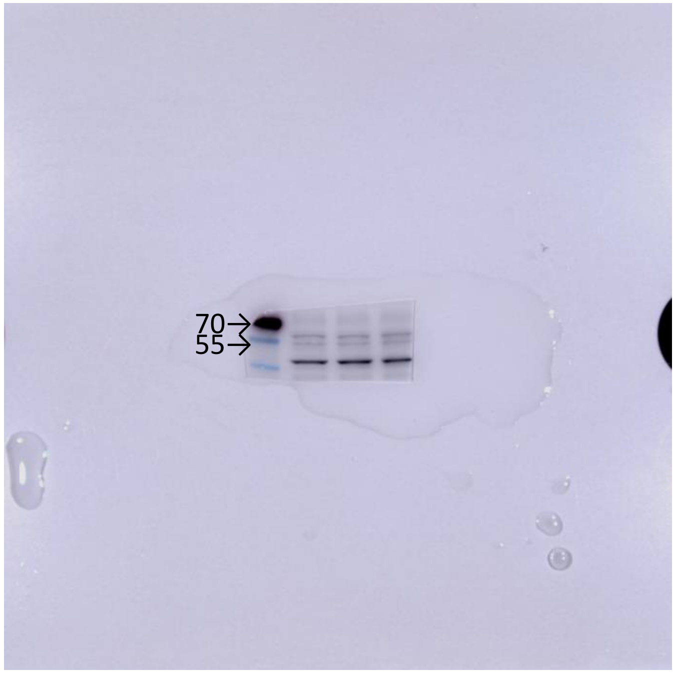

β-Actin 45kDa

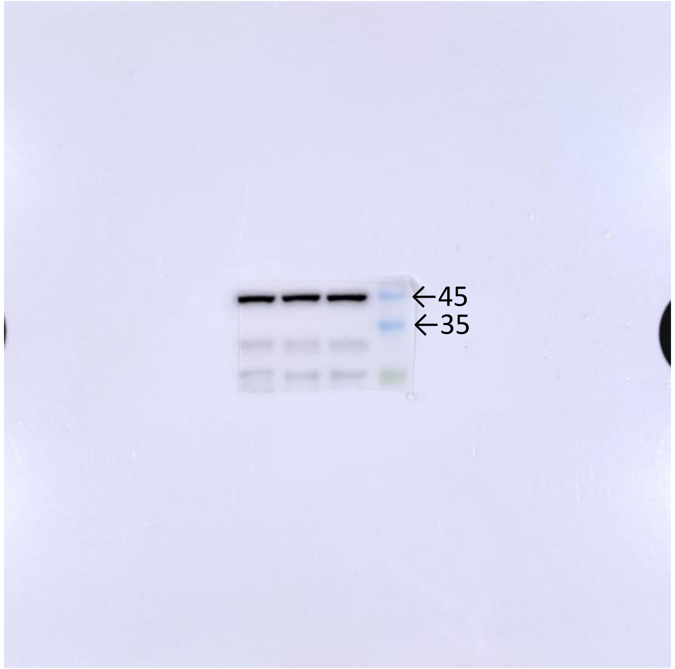

Fig.4.A

CD86 37kDa

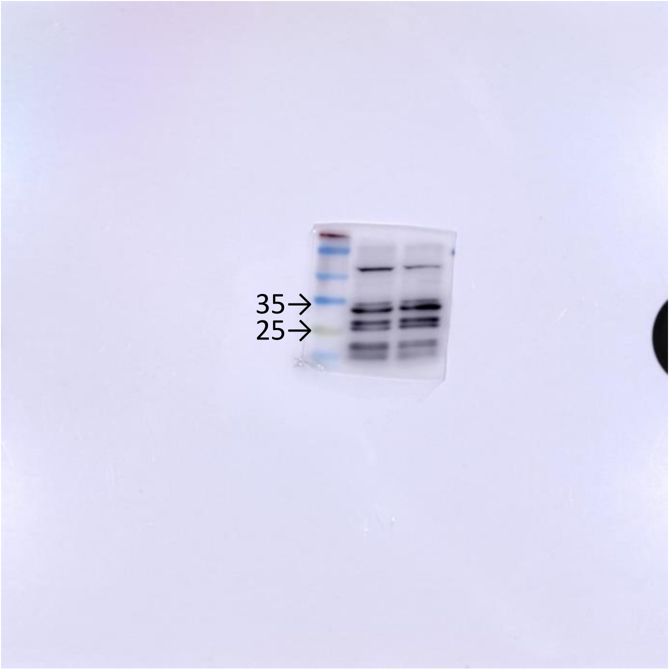

β-Actin 45kDa

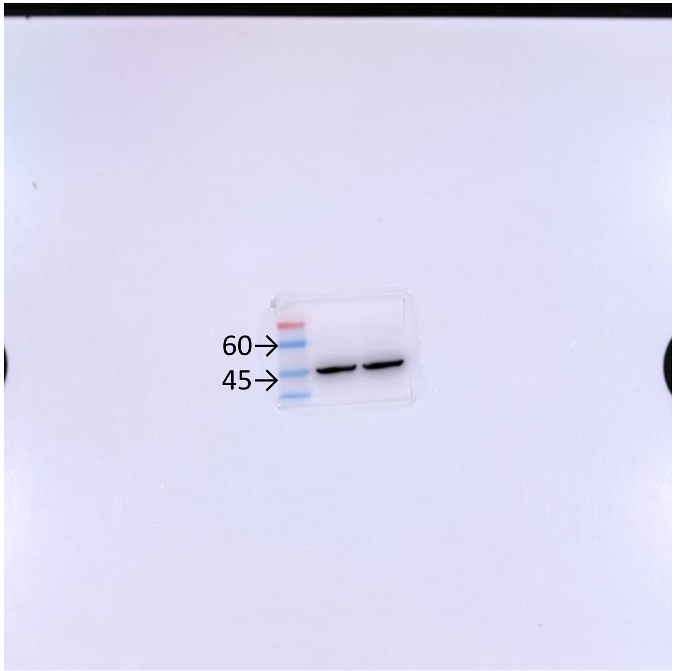

Fig.4.C

IL-1β 31kDa

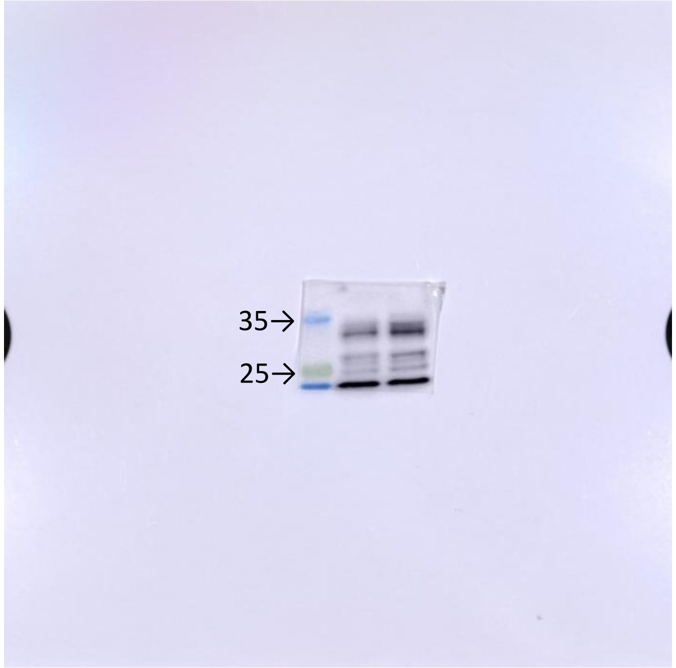

IL-6 23kDa

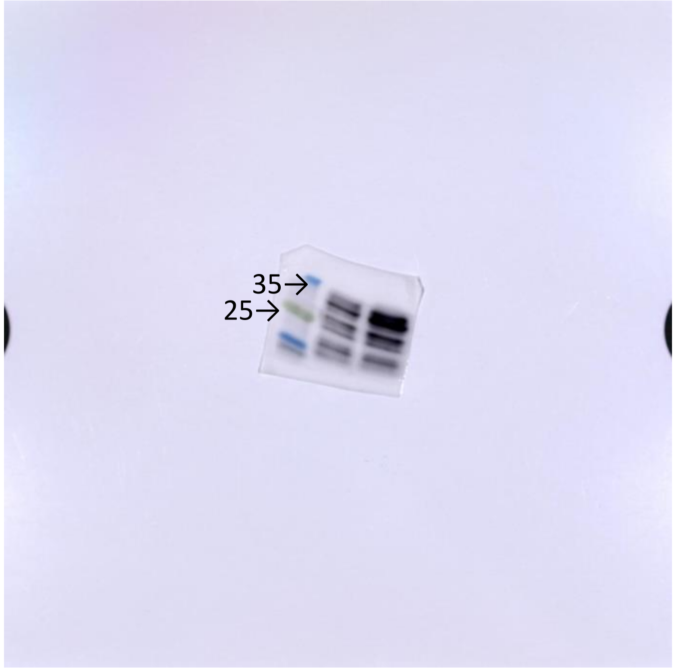

Fig.4.C

TNF-α 26kDa

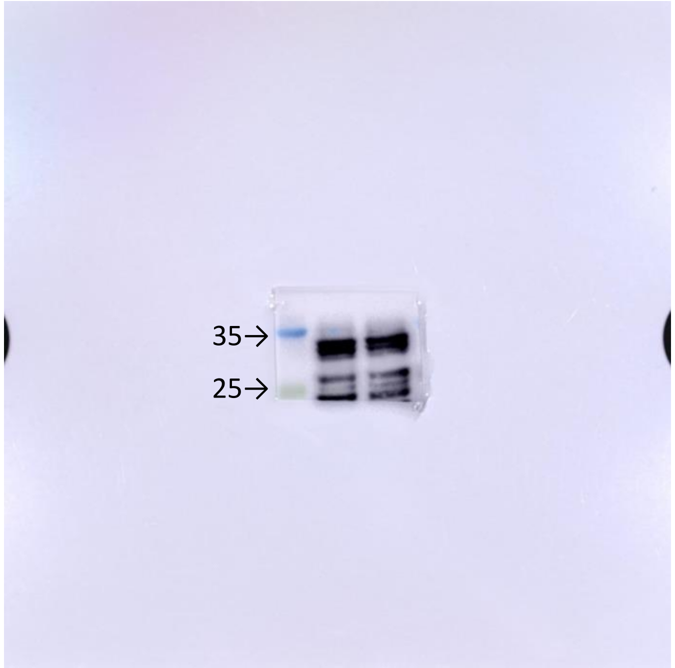

β-Actin 45kDa

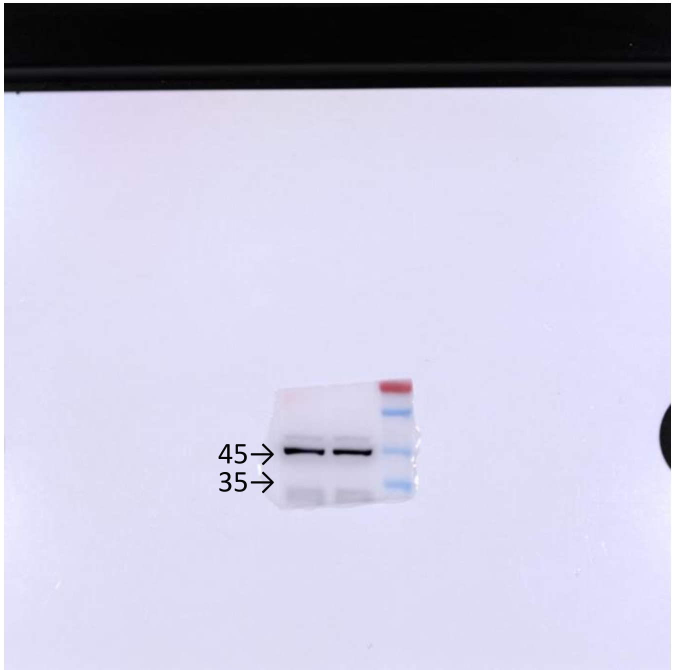

Supplement: Multimedia component 2 [file mmc2.pdf]
